# Multilocus, phenotypic, behavioral, and ecological niche analyses provide evidence for two species within *Euphonia affinis* (Aves, Fringillidae)

**DOI:** 10.3897/zookeys.952.51785

**Published:** 2020-07-23

**Authors:** Melisa Vázquez-López, Juan J. Morrone, Sandra M. Ramírez-Barrera, Anuar López-López, Sahid M. Robles-Bello, Blanca E. Hernández-Baños

**Affiliations:** 1 Museo de Zoología, Departamento de Biología Evolutiva, Facultad de Ciencias, Universidad Nacional Autónoma de México (UNAM), Apartado Postal 70-399. 04510 Mexico City, Mexico Universidad Nacional Autónoma de México Mexico Mexico

**Keywords:** *Euphonia affinis*, *Euphonia godmani*, independent evolutionary lineages

## Abstract

The integration of genetic, morphological, behavioral, and ecological information in the analysis of species boundaries has increased, allowing integrative systematics that better reflect the evolutionary history of biological groups. In this context, the goal of this study was to recognize independent evolutionary lineages within *Euphonia affinis* at the genetic, morphological, and ecological levels. Three subspecies have been described: *E. affinis godmani*, distributed in the Pacific slope from southern Sonora to Guerrero; *E. affinis affinis*, from Oaxaca, Chiapas and the Yucatan Peninsula to Costa Rica; and *E. affinis olmecorum* from Tamaulipas and San Luis Potosi east to northern Chiapas (not recognized by some authors). A multilocus analysis was performed using mitochondrial and nuclear genes. These analyses suggest two genetic lineages: *E. godmani* and *E. affinis*, which diverged between 1.34 and 4.3 My, a period in which the ice ages and global cooling fragmented the tropical forests throughout the Neotropics. To analyze morphometric variations, six morphometric measurements were taken, and the Wilcoxon Test was applied to look for sexual dimorphism and differences between the lineages. Behavioral information was included, by performing vocalization analysis which showed significant differences in the temporal characteristics of calls. Finally, Ecological Niche Models were estimated with MaxEnt, and then compared using the method of Broennimann. These analyses showed that the lineage distributed in western Mexico (*E. godmani*) has a more restricted niche than the eastern lineage (*E. affinis*) and thus we rejected the hypotheses of niche equivalence and similarity. Based on the combined evidence from genetic, morphological, behavioral, and ecological data, it is concluded that *E. affinis* (with *E. olmecorum* as its synonym) and *E. godmani* represent two independent evolutionary lineages.

## Introduction

The integration of genetic, morphological, ecological, and behavioral data in systematic studies provides information on the evolutionary history of species and their populations, allowing a better assessment of species limits (Cadena and Cuervo 2010, Köhler et al. 2010, Padial et al. 2010, Pavlova et al. 2014), as well as understanding the role of geographical and ecological factors on population divergence within species (Padial et al. 2010, Hernández et al., 2018). De Queiroz (2007) proposed that different types of data (e.g., morphological, ethological, ecological, molecular, etc.) are needed to determine operationally whether the lineages under study are evolving separately, and thus can be considered to represent different species. Species differentiation is affected by the time elapsed since the speciation event, a problem that should be considered in species delimitation studies. Padial et al. (2010) explained two ways to approach this problem: one is integration by congruence, and the other is integration by accumulation. In the first case (integration by congruence), taxonomists will consider two lineages as different species when there are concordant patterns of divergence among several taxonomic characters which result a full lineage separation. Meanwhile the integration by accumulation framework implies that divergences in any number of attributes (taxonomic characters) can provide evidence for the existence of a species, and in this case it is important to distinguish the group of characters (or even a single character) that promotes divergence and is reflected in the separation of lineages.

Species limits on birds have been studied using different approaches, including the use of morphological characters (Navarro-Sigüenza et al. 2001), coloration (Frith and Frith 1983, Rathbun et al. 2015), genetic variation (Olsson et al. 2013), songs (Sosa-López et al. 2013), and ecological niche modeling (Ruiz-Sánchez et al. 2015). In general, the objectives of these studies have been to resolve boundaries within species complexes and to evaluate subspecies recognized by taxonomic authorities such as the AOU (American Ornithology Union) and the IOC World Bird List (International Ornithology Committee).

DNA sequences have been useful to complement morphological and geographical information. Phylogenetics, molecular clocks, diversification rates, genetic populations and coalescence analyses have documented that geological complexity, heterogeneity of the environment, and climatic oscillations may have influenced patterns of genetic diversity, demography and divergence within species (de-Nova et al. 2012, Gu et al. 2013, Smith et al. 2014, Rodríguez-Gómez and Ornelas 2015). On the other hand, ecological niche modeling has provided information that supports the results of genetic studies on species delimitation (Raxworthy et al. 2007, Ruiz-Sánchez et al. 2015) and has helped discern whether speciation has been mediated by niche conservatism or ecological niche divergence (Brook et al. 2006, Wiens 2004, Wiens and Graham 2005). While vocal displays can be important prezygotic barriers to interspecific mating (Catchpole and Slater 2008), and many avian lineages have been discovered and, in part, diagnosed as distinct on the basis of differences in vocalization (Alström and Ranft 2003, Halley et al. 2017). However, with the application of the integrative taxonomy it has become possible to incorporate diverse information such as multilocus, morphological and ecological data to hypothesize species limits (Mckay et al. 2014, Minoli et al. 2014, Perez and Borges-Martins 2019, Venkatraman et al. 2018). This approach has been very useful even for cryptic species (Ramos et al. 2019).

**Figure 1.**
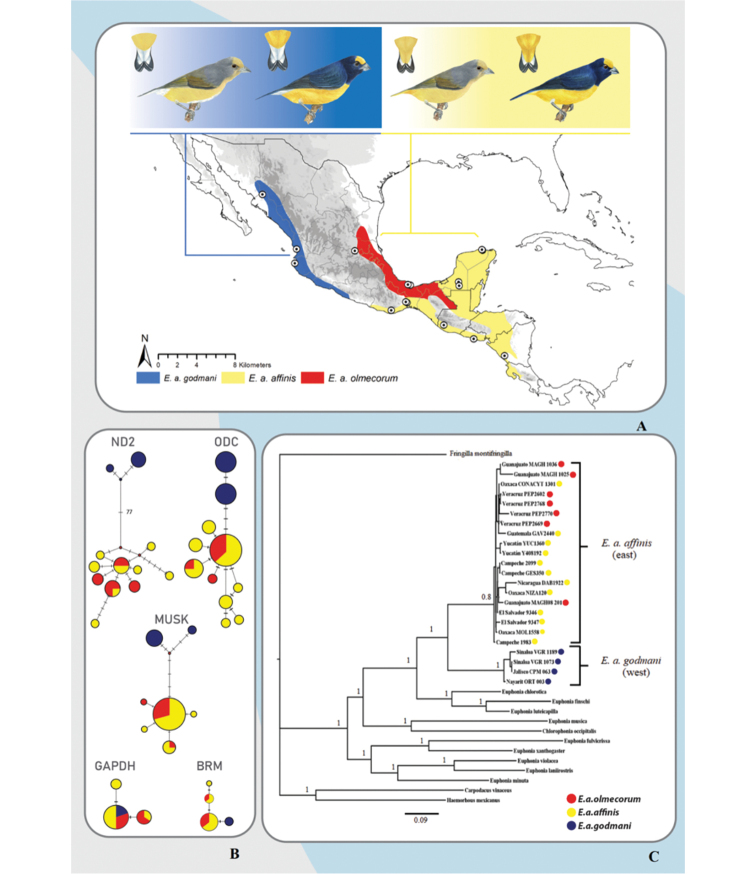
Geographic distribution and morphotypes of *Euphonia affinis*, sampling, phylogeny, and haplotype networks. **A** geographic distribution of *E. affinis*: in blue *E. a. godmani*, in yellow *E. a. affinis*, and in red *E. a. olmecorum* (Geographic distribution modified from NatureServe shapefile in ArcGIS, ArcMAP 10.2.2; Esri, Redlands, CA, USA). Tissue sampling locations are indicated by circles in the map. Plumage morphotypes of *E. a. godmani* (female and male) with white undertail coverts, and *E. a. affinis* (female and male) with yellow undertail coverts. The previously proposed subspecies *E. a. olmecorum* (not shown) is similar to *E. a. affinis*, but paler plumage in females and a purple-blue back in males have been reported. **B** haplotype networks obtained for the mitochondrial gene ND2 and the nuclear genes ODC, MUSK, GAPDH intron 11, and BRM intron 15. Samples from the western distribution, assigned as *E. a. godmani*, are shown in blue and from the eastern distribution, assigned as *E. a. affinis* are indicated in yellow, *E. a. olmecorum* in red. **C** bayesian Inference concatenated phylogeny of *E. a. godmani* (west) and *E. a. affinis*-*E. a. olmecorum* (eastern Mexico, Central America).

*Euphonia affinis* is a member of the family Fringillidae, subfamily Euphoniinae (Zuccon et al. 2012). *Euphonia affinis* appears to be phylogenetically related to *E. chlorotica, E. luteicapilla*, *E. finschi, E. plumbea*, *E. concinna*, and *E. trinitiis* (Isler and Isler 1987, Imfeld et al. 2020). Like all Euphoniinae, its distribution is restricted to the Neotropics. Specifically, *E. affinis* is a resident of the tropical lowlands from Mexico to Costa Rica (AOU 2003). In Mexico, the species is distributed along both slopes, from Sonora in the west and San Luis Potosi in the east, south to Central America (Howell and Webb 1995: fig. 1). Two subspecies are currently recognized based on geographical and morphological descriptions (AOU 1998). *Euphonia affinis godmani* Brewster, 1889 is endemic to western Mexico and is distributed from Sonora to central Guerrero. *Euphonia affinis affinis* Lesson, 1842 is distributed from eastern San Luis Potosi, southeastern Tamaulipas, Veracruz, Puebla and north-southwestern Oaxaca and the Yucatan Peninsula to Honduras, and on the Pacific Coast of Central America from Nicaragua to northwestern Costa Rica. The morphological characteristics that distinguish both subspecies are the subcaudal covert feathers, which are white in *E. affinis godmani* and yellow in *E. affinis affinis* in both males and females (Fig. 1). Dickerman (1981) described a third subspecies, *Euphonia affinis olmecorum*, based on differences in female coloration, which is paler than females of *E. affinis affinis*. *Euphonia affinis olmecorum* is distributed along the Gulf Coast of Mexico, from southeastern Tamaulipas and eastern San Luis Potosi to northern Chiapas (Hilty 2018); however, some taxonomic authorities do not recognize this subspecies, treating it as part of *E. affinis affinis* (Clement 2011). Currently, there are no studies of intraspecific limits for the species described in Euphoniinae. However, there is morphological and biogeographic evidence that the number of species is underestimated, with several species having wide ranges of distribution and more than one morphotype. Also, Imfeld et al. (2020) found genetic divergence between two subspecies of *E. xanthogaster* of similar magnitude to that between recognized species. Taken together, these observations indicate that species limits studies in Euphoniinae are needed.

In the present work, we applied integrative taxonomy to identify the independent evolutionary lineages within *Euphonia affinis* using four types of characters, multilocus genetic data, morphometric data, behavioral, and environmental niches. Based on the allopatric distribution of subspecies *E. a. affinis* (Eastern Mexico and Central America) and *E. a. godmani* (West of Mexico), as well as in the distinctive character of subcaudal feathers, we expect to recognize at least two independent evolutionary lineages that can be proposed to elevate at the species level.

Our goals were to: 1) obtain a phylogenetic hypothesis for *Euphonia affinis* subspecies using multilocus genetic data. 2) Associate the genetic variation and divergence times with historical geographic processes and barriers. 3) Describe the pattern of morphometric, behavioral, and environmental variation in *Euphonia affinis*, and associate it with genetic variation and phylogenetic relationships. Hence, our hypothesis is that multiple independent evolutionary lineages exist within the *Euphonia affinis* complex, and our objective is to define them with the integration of multilocus genetic data, morphometric, behavioral, and environmental data. Furthermore, we discuss a potential promotion of those lineages to species status.

## Materials and methods

### Taxon sampling and sequencing procedures

For the ingroup we used 19 tissues from *Euphonia affinis affinis* and four from *E. affinis godmani*; for the outgroup we obtained one tissue sample from *Chlorophonia occipitalis*, two from *Euphonia chlorotica*, one from *E. luteicapilla* and one from *Haemorhous mexicanus* (Suppl. material 1, Table S1). We completed our outgroup dataset with sequences from Genbank of *Euphonia chlorotica*, *E. finschi*, *E. lanirostris*, *E. minuta*, *E. musica*, *E. violacea*, *E. xanthogaster, Fringilla montifringilla, F. teydea, Carpodacus vinaceus* and *Haemorhous mexicanus* (Suppl. material 1, Table S2).

Genomic DNA was isolated using the Qiagen DNeasy^TM^ kit (Qiagen Inc., Valencia, CA, USA) following the manufacturer’s protocol. We amplified five molecular markers: one mitochondrial ND2 (NADH Dehydrogenase Subunit 2, Sorenson et al. 1999) and four nuclear genes ODC (Ornithine Decarboxylase, Allen and Omland 2003), GAPDH intron 11 (Glyceraldehyde-3-phosphate dehydrogenase, Friesen et al. 1997) , BRM intron 15 (BRM transcription regulatory protein, Marthinsen et al. 2009), and MUSK (Muscle, skeletal receptor tyrosine-protein kinase, Kimball et al. 2009). Technique specifications are given in Suppl. material 1, Table S3. Amplifications were done via PCR in 12.5 μl reactions. PCR and products were visualized on a 1% agarose gel. DNA sequencing was performed by the High-Throughput Genomics Unit service (University of Washington). We edited and aligned chromatograms in Sequencher v4.8 (GeneCodes Corporation, Ann Arbor, MI). All sequences were deposited in GenBank; with the accession numbers ND2: MT452146–MT452170, ODC: MT452191–MT452215, GAPDH: MT452124–MT452145, BRM: MT452098–MT452123 and MUSK MT452171–MT452190. (Also included in https://github.com/almamelisa/Euphonia-affinis-complex).

### Phylogenetic analysis

We constructed the alignment of each gene using the CLUSTAL IW (Thompson et al. 1994) function in BIOEDIT (Hall 1999). Then, we used JModelTest 0.1.1 (Posada 2008) to estimate evolutionary models for each molecular marker. We conducted phylogenetic analyses in MrBayes v3.2.3 (Huelsenbeck and Ronquist 2003) for Bayesian Inference (BI), using a partitioned dataset including the four nucleotide genes ODC, MUSK, GAPDH, and BRM; ND2 was partitioned by codon position (see Table 2). For the BI, we ran the process for 10 million generations, sampling every 1,000 generations. We examined the convergence of the chains with Tracer v1.6 (Rambaut and Drummond 2013) and discarded the first 25% of generations as burn-in.

### Genetic diversity and structure

To resolve heterozygotes in nuclear sequences, we used a Bayesian approach in PHASE v 2.1 (Stephens et al. 2001), selecting the pairs of haplotypes with a posterior probability higher than 0.90. Then, we used DnaSP v5.0 (Librado and Rozas 2009) to estimate the number of haplotypes (H), haplotype (Hd) nucleotide diversities (π), and Fst. Genetic distances were obtained with MEGA 5 (Tamura et al. 2013). Finally, we obtained the haplotype network for each gene with the median-joining algorithm using Network v4.6 (Bandelt et al. 1999) through DnaSP.

### Tests of divergence times

Divergence times were estimated from the multilocus dataset with three genes, the mitochondrial gene ND2, and the two nuclear genes with sequences for all samples and outgroups (ODC and GAPDH) using Beast v1.8 (Drummond et al. 2013). As point calibration, we used a secondary dating based on the divergence between Fringillidae and the New World nine-primaried Oscines 17.1104 My with a 95% HPD of 14.7743–19.6278 calculated by Oliveros et al. (2019). We assigned a Normal distribution to the secondary dated point. Additionally, we defined partitions with different evolutionary rates corresponding to each gene fragment (ND2 = 0.029 s/s/My, ODC = 0.0014 s/s/My and GAPDH = 0.0012 s/s/My), based on Lerner et al. (2011). We used a Yule model as a tree prior (Gernhard 2008). For the molecular clock model, we selected the normal relaxed molecular clock following Drummond et al. (2006) and Li and Drummond (2012). We performed 20 million generations, sampling every 1,000 and corroborating the appropriate effective sample size (ESS > 200) with TRACER v1.6 (Rambaut and Drummond 2013). Finally, in TREE ANNOTATOR v 1.8.0 (Rambaut and Drummond 2013), we did a burn-in of 5,000 trees and produced the maximum clade credibility tree with 95% highest probability densities. The tree was visualized with FigTree v1.4.2 (Rambaut 2014).

### Morphometrics

Six morphometric measurements of 355 specimens (233 males and 122 females) were taken from the following collections (see Suppl. material 2, also included in https://github.com/almamelisa/Euphonia-affinis-complex): Museo de Zoología Alfonso L. Herrera (UNAM), Colección Nacional de Aves-Instituto de Biología (UNAM), Moore Laboratory of Vertebrate Zoology, American Museum of Natural History, Louisiana Museum of Natural History, Academy of Natural Sciences, Museum of Comparative Zoology, and Delaware Museum of Natural History. The morphometric measurements (following the recommendations of Baldwin et al. 1931) were: bill length (**BL**, from the upper base of the bill to the tip of the upper mandible), bill width (**BW**), bill depth (**BD**, from the upper mandible to the base of the bill at the distal edge of the nostrils), wing chord (**WC**, distance from the carpal joint the tip of the longest primary), tarsus length (**TL**), and tail length (**TLE**, distance from the uropygial gland to the tip of the longest rectrix). All measurements were taken only by the first author to avoid bias in the process using a dial caliper with a precision of 0.1 mm, except for tail length, which was taken with a millimeter ruler and in three independent events. To obtain our final data set we averaged the three independent events for every measure. Since both our molecular phylogenetic results and our analysis of the previously proposed plumage color differences do not provide evidence of *E. affinis olmecorum* as an independent evolutionary lineage, we decided to analyze morphometric variation, vocalization, and ecological niche only between *E. affinis affinis* and *E. affinis godmani*.

The normality was tested with the Shapiro-Wilk test of normality in R (R Core Team 2017). Since, the normality was rejected in all except one of the groups, we evaluated the sexual dimorphism with the Unpaired Two-Samples Wilcoxon Test, also with basic R functions. We obtained significance differences between male and females in three variables WC, TLE, and BD (see results), so we evaluated these variables differences between the lineages in a separated way for males and females with the Unpaired Two-Samples Wilcoxon Test. The rest of the variables were evaluated jointly for both sexes and also with the Wilcoxon test. With the previous arrangement, we also did a Principal Component Analysis based on the correlation matrix with the R package Factominer (Lê and Husson 2008). Graphs were generated in R package Factorextra (Kassambara and Mundt 2016). All the scripts and input data are in https://github.com/almamelisa/Euphonia-affinis-complex.

### Vocalization

We obtained 19 recordings of *Euphonia affinis* calls from the Xeno-Canto (XC; http://www.xeno-canto.org) open access database. We used only call recordings in which the subspecies was identified and in which *Euphonia* was identified as the foreground species. We visualized and measured spectrograms of these recordings using the Raven Pro 1.6 software (Cornell University, Ithaca, NY). We visually inspected the spectrograms, and from each recording we selected one call section that did not overlap with any background vocalizations or other sounds. In recordings where more than one call variant occurred (for example, variants with differing number of notes), we selected one of the most frequent type. The most common call type for this species consists of a short series (2 to 4 notes) of whistled notes with decreasing pitch. Since recording conditions were not standardized, we only took frequency and duration measurements, which are not heavily affected by distance. We measured low and high frequencies (LowFreq and HiFreq), change in frequency (DeltaF), duration of call (DeltaT), number of notes (Notes) and emission rate (Speed; number of notes divided by duration). All measured variables were rescaled by *log* transforming them.

Unpaired Two-Samples Wilcoxon Test were carried out on individual variables to test for differences between the two groups. We also performed a principal component analysis (PCA) to explore the relation between the two groups in multivariate space. All the scripts and input data are in https://github.com/almamelisa/Euphonia-affinis-complex.

### Ecological niche modeling and paleodistribution

The georeferenced records were obtained from the specimens used in the morphometric and genetic analyses, 102 for *E. affinis affinis* and 29 for *E. affinis godmani*. To define the M area (accessibility area; *sensu* Soberón & Peterson, 2005) for each evolutionary lineage herein identified, we plotted the record points onto the biogeographic provinces of the Neotropical region (Morrone 2014) and chose the provinces that matched the record points for both lineages, using the shapefiles provided by Löwenberg-Neto (2014). Such considerations assumed that these regions may define the accessible historical area and specific restriction region for each lineage (Svenning and Skov 2004).

For the first explorative analysis, we used the 19 bioclimate layers from WorldClim and assessed which variables were the most important for the model, according to the Jackknife test calculated in MaxEnt (Royle et al. 2012). In a second modeling exercise, we generated the species models using those non-correlated (r < 0.8) environmental variables in combination with the most relevant environmental variables identified in the first approach. According to previous published works (Ortega-Andrade et al. 2015, Hernández et al. 2018), these additional steps allowed us to reduce overfitting of the generated distribution models, minimizing the collinearity problems among variables (Dormann et al. 2013). Pearson correlation test among bioclimatic variables was performed in R with the basic commands. Final models were performed considering only those 12 climatic variables: BIO3 = Isothermality (BIO2/BIO7) (* 100), BIO5 = Max Temperature of Warmest Month, BIO6 = Min Temperature of Coldest Month, BIO7 = Temperature Annual Range (BIO5-BIO6), BIO8 = Mean Temperature of Wettest Quarter, BIO9 = Mean Temperature of Driest Quarter, BIO10 = Mean Temperature of Warmest Quarter, BIO14 = Precipitation of Driest Month, BIO15 = Precipitation Seasonality (Coefficient of Variation), BIO16 = Precipitation of Wettest Quarter, BIO 18 = Precipitation of Warmest Quarter, and BIO 19 = Precipitation of Coldest Quarter. The climatic layers were used in ascii format and ~ 1 km resolution, and they were cut to the shape of the M area using the R package Raster (Hijmans 2019). We generated the final Ecological Niche Model (ENM) for each lineage using 75% of the record points as training data and 25% as testing data. We performed 25 replicates, 500 iterations, with 0.00001 as the convergence limit and 0.5 prevalence.

To evaluate the models we calculated the partial ROC (Receiver Operating Characteristic) in the web tool Niche Tool Box (https://shiny.conabio.gob.mx:3838/nichetoolb2/), the parameters were 0.05 proportions of omission, 50 random points percentage and 500 iterations. Also, in MaxEnt, we made four models projections one in the M area of each lineage and the remaining three were made to obtain the paleodistribution; we projected the ENM in the last maximum glacial period (~ 22,000 years ago) considering two general circular models: MIROC-ESM (Hasumim and Emori 2004) and CCSM (Collins et al. 2004). We also projected the ENM onto the last interglacial period (~120,000–140,000) (Otto-Bliesner et al. 2007). Finally, using the R packaged Ecospat (Di Cola et al. 2017) we evaluated the ecological overlap between the lineages, to quantify equivalence and similarity among groups using the Broennimann´s method (Broennimann et al. 2012). It consists of three steps: the first one is to calculate the density of occurrences and environmental factors across the axes of the environmental principal component analysis; the second is to evaluate the superposition niche along the gradient of multivariate analysis. Finally, the Schoener’s D observed (Schoener 1968) and the statistical similarity *I* observed (Warren et al. 2008) are compared with the 100 repetitions of randomly generated simulated values for *D* and *I* (Warren et al. 2008, Broennimann et al. 2012). This final step consists in testing two hypotheses, the equivalence and similarity among groups. The hypotheses of niche equivalence and similarity are rejected if the empirically observed *D* and *I* values are significantly different from the values expected from the pseudoreplicates. All the scripts and input information are in https://github.com/almamelisa/Euphonia-affinis-complex.

## Results

### Genetic diversity and phylogenetic analyses

The multilocus dataset analyses revealed a well-supported monophyly for the *Euphonia affinis* complex and recovered two main phylogroups: one included the samples from western Mexico (*E. affinis godmani*) and the other comprised samples from eastern Mexico and Central America (*E. affinis affinis* and *E. affinis olmecorum*) (Fig. 1). The sister group of *E. affinis* complex was the clade including *E. chlorotica*, *E. luteicapilla*, and *E. finschi*. As shown in Table 2, the genetic distances between *E. a. affinis* and *E. a. godmani* have values similar to genetic distances between the other species.

The haplotype network obtained with ND2 sequences showed two geographically structured haplogroups: a western group and an eastern-CA group (Fig. 1), respectively, with two haplotypes of *E. affinis godmani* and 10 that included samples of *E. affinis affinis* and *E. affinis olmecorum* separated by 77 permutations. ND2 had the highest total haplotypic diversity (see Table 1). These same two groups were obtained for ODC, MUSK, and BRM genes. The only exception was the GAPDH network, which did not recover these geographically structured groups. Also, in Table 1 we show the results of nucleotide diversity, Tajima’s *D*, nucleotide composition, molecular evolutionary models, parsimony informative sites, monomorphic sites, and the alignment base pairs.

**Table 1.** Diversity indices, nucleotide content, evolution model, variation sites, and alignment base pairs of mtDNA and nDNA.

Gene	H-A	Hd			Pi			D	NC				MEM	PIS	MS	Alignment BP
		Hd	Σ	SD	Pi	σ	SD		%T	%C	%A	%G				1049
**ND2**	12	0.95	7E-04	3E-02	0.019	7E-05	8E-03	-0.01*	26	32.5	31.6	10.4	TVM+G	358	598	(997–1041)
**ODC**	7	0.56	6E-03	8E-02	0.004	9E-07	9E-04	-0.67*	36.6	16.9	27.3	19.1	HKY	10	535	556
**MUSK**	11	0.86	1E-03	4E-02	0.004	3E-07	5E-04	-0.70*	32.6	16.8	30.5	20.3	TPM1uf	8	476	500
**GAPDH**	3	0.17	5E-03	7E-02	0.0006	1E-07	7E-02	-1.13*	25.4	19.9	21	33.7	HKY	1	262	280
**BRM**	4	0.5	5E-03	8E-02	0.002	2E-06	3E-04	-0.30*	34.6	12.9	34.6	17.9	HKY	2	288	304

H-A number of haplotypes and alleles. Hd haplotype diversity. Pi nucleotide diversity. D’Tajima. NC nucleotide composition. MEM Molecular evolution model. PIS Parsimony informative Sites. MS monomorfic sites. Alignment BP. For ND2 in () range of sequence large. P< 0.01*

**Table 2.** Genetic distances.

	ND2				ODC				MUSK				GAPDH				BRM			
	**1**	**2**	**3**	**4**	**1**	**2**	**3**	**4**	**1**	**2**	**3**	**4**	**1**	**2**	**3**	**4**	**1**	**2**	**3**	**4**
**1**																				
**2**	0.11^**^				0.01^**^				0.01^**^				0.00^**^				0.01^**^			
**3**	0.14^*^	0.17^*^			0.01^**^	0.01^**^			0.02^**^	0.02^**^			0.05^**^	0.05^**^			0.16^*^	0.06^*^		
**4**	0.15^*^	0.16^*^	0.09^*^		0.01^**^	0.01^**^	0.01^*^		–	–	–		0.05^**^	0.05^*^	0.00^**^		0.07^*^	0.07^*^	0.07^*^	
**5**	0.14^*^	0.15^*^	0.09^*^	0.03^*^	0.01^**^	0.01^**^	0.01^*^	0.01^**^	–	–	–	–	0.121^*^	0.121^*^	0.115^*^	0.115^*^	–	–	–	–

1. *E. a. affinis* 2. *E. a. godmani* 3. *E. chlorotica* 4. *E. luteicapilla* 5. *E. finschi*. P <0.005** P < 0.05*

### Divergence times

**Figure 2.**
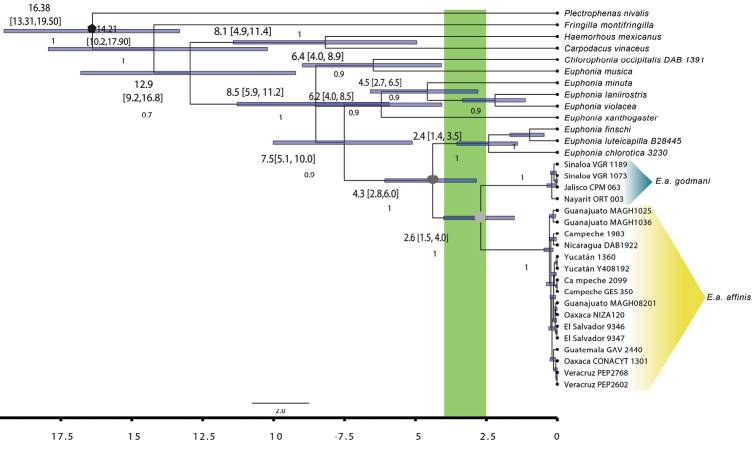
Ultrametric phylogenetic tree obtained by BEAST using ND2, ODC, and GAPDH concatenated matrix. The rhombus node represents the calibration point 17.1104 My with a 95% HPD of (14.7743, 19.6278) (see methods), dark gray circle node represents the *E. affinis* origin and light gray circle node represents the break between *E. a. godmani* and *E. a. affinis*. Above the branch the diversification dates (My) and in brackets the 95% HPD. Below branch the number indicated the posterior probability. The green area corresponds to the period when lowland dry forests had a greater expansion in Western Mexico.

*Euphonia affinis godmani* and *E. affinis affinis* split 2.6 Mya (1.5–4.0 Mya, 95% HPD), during the Late Pliocene-Early Pleistocene (Fig. 2). According to our analyses, the family Fringillidae is divided in three subfamilies: the oldest, Fringillinae, originated 14.21 Mya (10.2–17.9 Mya, 95% HPD, Highest Posterior Density), the split between Carduelinae and Euphoniinae was 12.9 Mya (9.2–16.8, 95% HPD), the Euphoniinae origen was 8.5 Mya (5.9–11.2 Mya, 95% HPD), and Carduelinae diverged 8.1 Mya (4.9–11.4 Mya, 95% HPD. Our estimate for the split between Fringillidae and *Plectrophenas nivalis* was 16.38 Mya (13.3–19.5 Mya, 95% HPD) while our point of calibration was 17.1104 Mya with a 95% HPD (14.7743–19.6278; Oliveros et al. 2019). Also, our results for the Fringillinae node age and the split between Carduelinae and Euphoniinae are consistent with the ages calculated for *Euphonia* and *Chlorophonia* phylogeny in Imfeld et al. (2020). However, there are also some differences between the ages calculated by us and by Imfeld et al. (2020), since they calculated that the split between *E. affinis* and *E. luteicapilla* was less than 1 Mya, whereas we calculated that the split between *E. affinis* and the rest of Euphonias was 4.3 Mya ago, and *E. affins* seems to be a sister group of *E. chlorotica*, *E. luteicapilla* and *E. finschi*.

### Morphometrics

**Figure 3.**
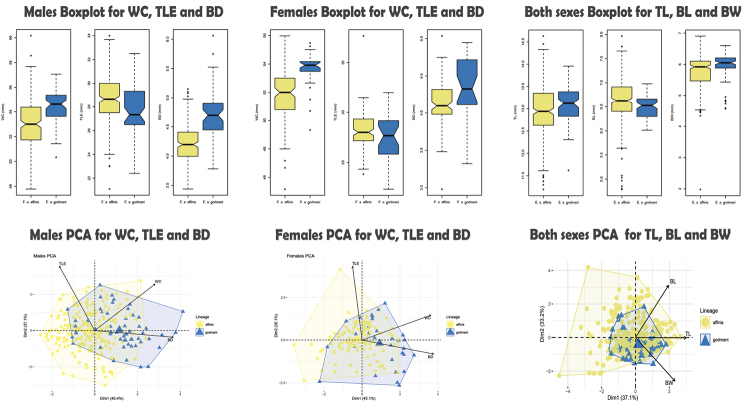
Morphometric analyses results. **A)** Females boxplots and PCA for WC, TLE, and BD morphometric characters. **B)** Males boxplot and PCA for WC, TLE, and BD morphometric characters. **C)** Boxplot and PCA for TL, BL, and BW. WC, TLE, and BD characters were analyzed by separated sex, because the analyses indicated sexual dimorphism (see results and Table 3). Bill length (BL, from the upper base of the bill to the tip of the upper mandible), bill width (BW), bill depth (BD, from the upper mandible to the base of the bill at the distal edge of the nostrils), wing chord (WC, distance from the carpal joint the tip of the longest primary), tarsus length (TL), and tail length (TLE, distance from the uropygial gland to the tip of the longest rectrix).

A total of 355 specimens was analyzed, of which 180 were males and 97 females of *E. affinis affinis*, and 53 males and 25 females of *E. affinis godmani.* Morphometric sexual dimorphism was found in three variables: TLE (Tail Length), WC (Wing Chord), and BD (Bill Depth) (Fig. 3, Table 3). Males showed statistically significant differences between *E. affinis godmani* and *E. affinis affinis* for these three characters, whereas females differed significantly in only two characters (WC and BD, Fig. 3). For the variables TL (Tail Length), BL (Bill Length), and BW (Bill width) we found significant differences between both groups (Fig. 3, Table 3). PCA analyses for males showed an 83.5% proportion of variance explained for two principal components, while females showed an 80.2% proportion of explained variance explained. Both Component plots showed that *E. affinis godmani* was distributed in quadrant 2, while *E. affinis affinis* had a wide distribution. The dispersion plot showed a partial overlap between *E. affinis affinis* and *E. affinis godmani*, but wing chord and bill depth were clearly differentiated in boxplots. These were also the two most important variables included in the first principal component in both sexes, according to the eigenvectors (Fig. 3).

PCA analyses for both sexes showed that the first two principal components explained a large proportion of the variance (70.37%, Table 3). Contrary to the previous graphs, both sex component plots show a total overlap between the two lineages.

**Table 3.** Median, Unpaired Two-Samples Wilcoxon Test to evaluate differences between lineages and sexes, p-value < 0.05 (in bold).

Between lineages
	Both sexes	PC1	PC2	PC3
	affinis	godmani	p-value	37.1%	33.2%	29.6%
TL	12.93(11.1–14.6)	13.12 (11.6–13.9)	3.10E-02	0.74	-0.0047	-0.67
BL	6.62(4.7–7.9)	6.52(6.0–6.9)	1.36E-04	0.48	0.76	0.42
BW	5.92(1.9–6.9)	6.05(4.5–6.6)	1.42E-03	0.57	-0.63	0.51
Females	PC1	PC2	PC3
	affinis	godmani	p-value	45.96%	34.74%	19.28%
WC	52.16 (46.6–55.97)	53.83 (49.3–55.2)	1.15E-04	0.77	0.4	-0.48
TLE	28 (23.8–37.6)	27.66 (22.3–32.0)	0.227	-0.24	0.93	0.26
BD	4.11 (3.4–5.0)	4.32 (3.3–4.9)	5.68E-03	0.84	-0.09	0.52
	Males	PC1	PC2	PC3
	afifnis	godmani	p-value	47.7%	35.5%	16.8%
WC	53(47.7–60.1)	54.61(51.4–57.0)	7.64E-08	0.7	0.56	-0.41
TLE	28.66(21.0–34.0)	27.33 (22.4–32.5)	3.27E-03	-0.4	0.85	0.31
BD	4.19(3.5–5.1)	4.68(3.7–6.6)	1.43E-13	0.87	-0.06	0.48
Sexual dimorphism
E. a. affinis	E. a. godmani
	females	males	p-value	Females	males	p-value
TL	13.017(11.1–14.6)	12.908(11.3–14.1)	0.11	13.213(12.3–13.9)	11.6–13.9)	0.43
BL	6.607(4.7–7.6)	6.633(5.0–7.9)	0.09	6.537(6.0–6.9)	6.523(6.0–6.9)	0.60
BW	5.947(4.5–6.9)	5.914(1.9–6.6)	0.99	6.043(4.5–6.4)	6.06(4.5–6.6)	0.81
WC	52.167(46.6–55.9)	53(47.7–60.1)	3.19E-04	53.833(49.3–55.2)	54.613(51.4–57.0)	6.22E-03
TLE	28(23.8–37.6)	28.667(21.0–34.0)	1.48E-02	27.667(22.3–32–0)	27.333(22.4–32.5)	0.78
BD	4.117(3.4–5.0)	4.1985(3.5–5.1)	0.08	4.327(3.3–4.9)	4.683(3.7–6.0)	4.40E-03

### Vocalization

**Figure 4.**
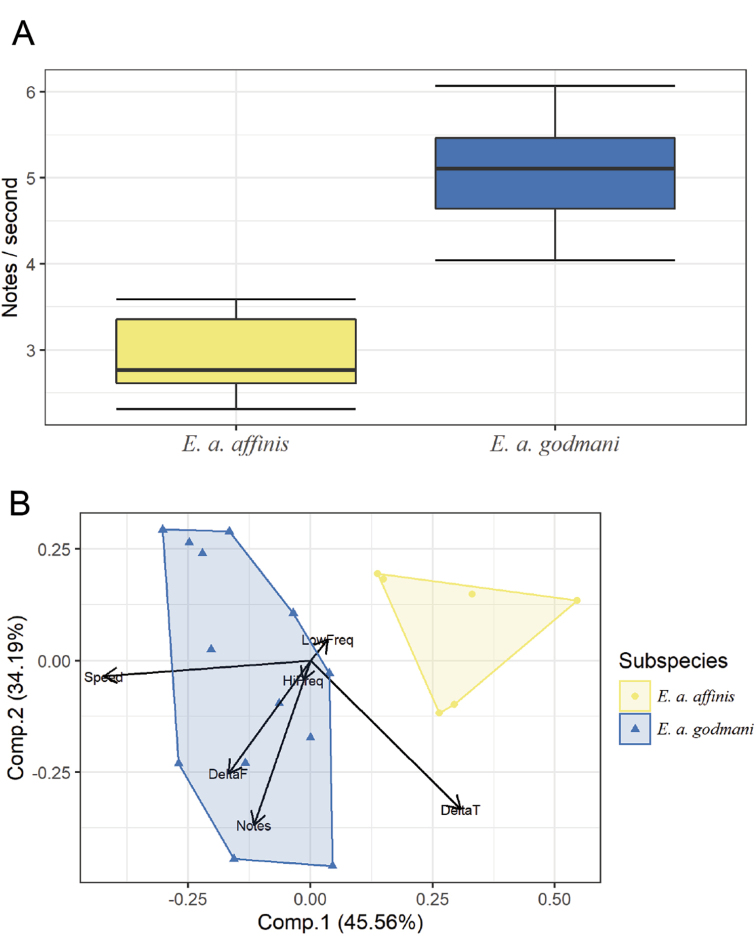
Vocalization analysis. Boxplot of note emission rate **A** and PCA of measured vocal characters **B** Calls differ between the two groups in temporal structure, but not in frequency or number of notes.

None of the frequency variables measured differed significantly between *godmani* and *affinis* groups (*N* =19). On the other hand, we found that the emission rate of *E. a. affinis* is much lower than that of *E. a. godmani*, on average 2.9 notes/s versus 5.09 notes/s. These differences are statistically significant (*W* = 0, *p* < 0.001). The first two Principal Components together explain 78.75% of variance. The first PC separates both groups unambiguously (Fig. 4), and has a highly positive correlation with call duration, as well as a highly negative correlation with emission rate (notes per second).

### Distribution modeling, paleodistribution, and ecological niche overlap

Our models obtained a high mean value for AUC (Area Under the Curve) ratio values and statistically significant, 1.68 for *E. affinis affinis* and 1.81 for *E. affinis godmani* (***P < 0.05), this indicates a good fit of ENM’s. According to the Jackknife test and contribution variables obtained by MaxEnt, the most important variable for *E. affinis affinis* model was BIO 15 (precipitation seasonality), and the variable BIO8 (mean temperature of wettest quarter) for *E. affinis godmani*. We present the ENM predictions in four levels in Fig. 4; the first ones are the present predictions for the two lineages and the overlap between them, in general, both models predicted the previously known area of distribution for both species (Fig. 4), with an overlap in the West Pacific coast. In the second level, we observed that *E. affinis godmani* has a limited ability to predict its ecological niche in the geographic areas where *E. affinis affinis* is distributed, while *E. affinis affinis* projected its ecological niche on a large geographic area of distribution for *E. affinis godmani*.

The third part is the projection of the models in Last Glacial Maximum conditions (LGM 21–18,000 years ago), for *E. affinis affinis* showing a reduction in their environmental suitability along the present distribution, with predictions in areas like the Yucatan Peninsula and the western coast of Mexico with a gap at the western coast of the Tehuantepec Isthmus, unlike Present predictions where Central America has only small patches with predictions for *E. affinis affinis* (Fig. 4). For *E. affinis godmani* we found predictions in the western coast of Mexico, with a large gap between the central Mexican Coast west and the western coast of the Tehuantepec Isthmus, it also has a small patch prediction in the Yucatan Peninsula. The fourth part is the Last Interglacial (~ 120,000–140,000 years ago), for both lineages, the areas with high environmental suitability increased with respect to LGM, for *E. affinis affinis* it including the western Yucatan Peninsula, the Central western Mexican coast, and the Tehuantepec Isthmus to the western coast of Central America. For *E. affinis godmani*, the prediction areas are the West Mexican coast and the western Yucatan Peninsula.

**Figure 5.**
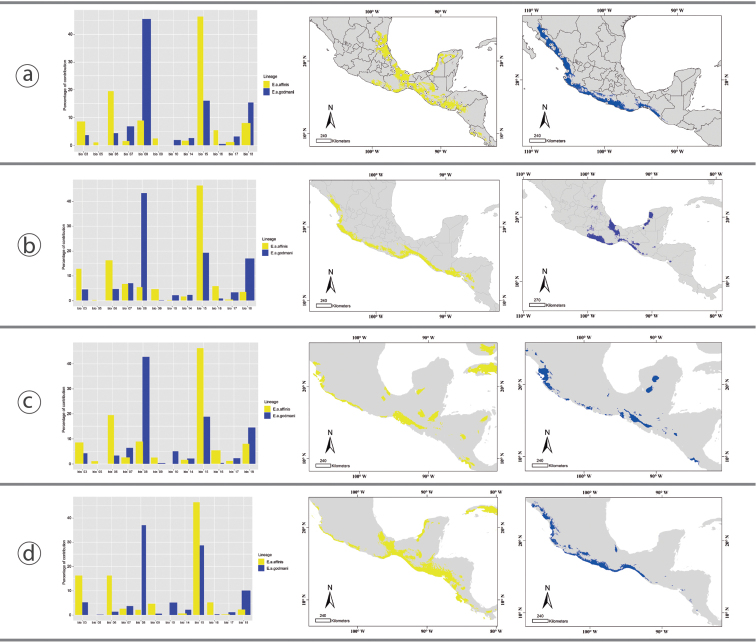
Ecological niche modelling and its projection in the geographic areas for *E. a. affinis* (yellow) and *E. a. godmani* (blue). In all four panels (a-d), the contribution values of each environmental variable of ENM’s is illustrated in the left and the projection of the Ecological niche conditions in the geographic distribution area is shown in the maps. **a** Ecological Niche projected in the current geographic distribution area of *E. affinis* and *E. a. godmani*. **b** ENM’s projected into the geography for each lineage. **c** ENM of *E. a. affinis* and *E. a. godmani* projected in the Last Maximum Glacial ecological conditions. **d** ENM of *E. a. affinis* and *E. a. godmani* projected in the Last Inter Glacial ecological conditions.

The results of ecological overlap for the environmental PCA exhibit a large niche of *E. a. affinis*, while *E. a. godmani* exhibits an ecological niche compaction. A total variance of 83.67% is explained for the three principal components, with 41.04% for PC1, 29.886% for PC2 and 12.733% for PC3 (Fig. 5). The *D* and *I* statistic observed values were close to zero 0.01174013 and 0.05643784 respectively. We can reject the niche equivalency because the *D* observed value does not fall within the density of 95% of the random simulated values, however we obtained a no significant *p* value (0.9901). In contrast, the niche similarity test between *E. affinis affinis* and *E. affinis godmani* shows that they are less similar than expected by chance (Fig. 5), since there is not significant climatic niche conservatism (p = 0.31683, p = 27723) between them. With this evidence we reject the niche conservatism hypothesis between *E. a. affinis* and *E. a. godmani*, so we can say that the niches are divergent (Warren et al. 2008, 2010; Broennimann et al. 2012).

**Figure 6.**
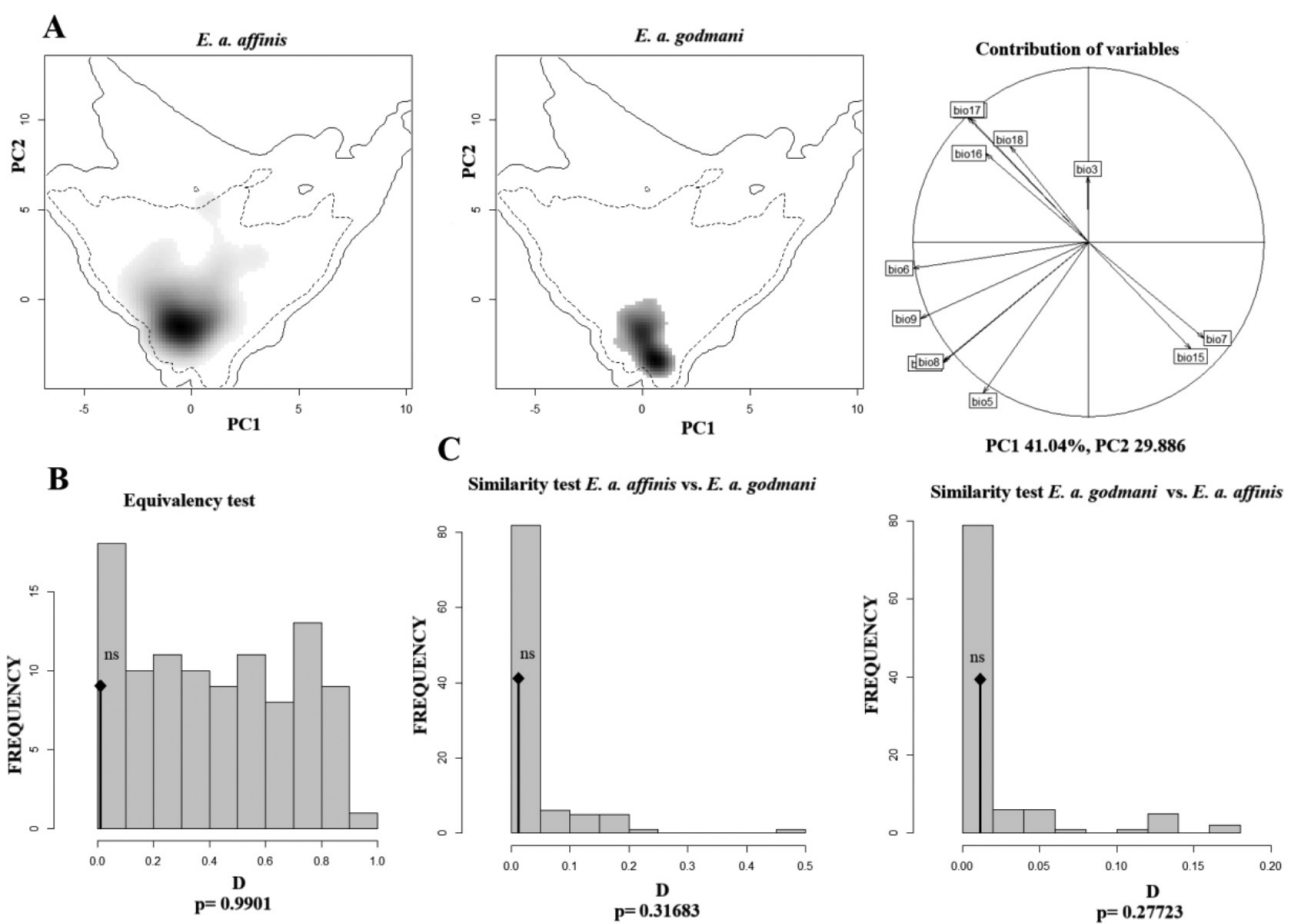
Equivalence and similarity tests in environmental space for *E. a. affinis* and *E. a. godmani*. **A** PCA of Ecological niche for of *E. affinis* lineages and the variables contribution to the analyses. The gray gradient indicates the density of the occurrences of the lineages, and the dashed and solid line indicates the 50% and 100% of the environmental background **B** graphical results of the equivalency tests comparing the two lineages. For both tests (equivalence and similarity) we only presented values for the D metrics. For all graphs the D observed values of the overlap niche analyses are present with the black diamond. The p value is showing in each graphic, all of them not significant for these analyses **C** graphical results of the similarity test comparing the two lineages in both directions (*E. a. affinis* vs. *E. a. godmani* and vice versa), ns = Not significant, p > 0.05.

### Descriptions

#### Euphonia affinis

Taxon classificationAnimaliaPasseriformesFringillidae

(Lesson, 1842) stat. nov.

F037708E-FA1B-51D7-A987-9399E74EE2D5

Tanagra affinis Lesson 1842, *Rev. Zool*. 5: 175.
Tanagra affinis affinis ; Miller et al. 1957, *Cooper Ornithol. Soc. Pac. Coast Avifauna* 33: 298.
Euphonia affinis affinis ; Dickerman, 1981, *Occ. Pap. Mus. Zool. Louisiana State. Univ*. 59: 3.
Euphonia affinis olmecorum Dickerman, 1981, *Occ. Pap. Mus. Zool. Louisiana State. Univ*. 59: 4, syn. nov.

##### Morphological characterization.

Males. Yellow forehead, back black with bluish to violet glow, black throat, yellow from chest to belly, yellow subcaudal coverts feathers (Hilty 2018). Morphometric characters, wing chord 52.167 mm, tail length 28.667 mm and bill depth 4.1985 mm.

Females. Forehead olive-yellow and gray, olive-green back. The throat is olive-yellow, with a yellow belly, subcaudal coverts feathers also in yellow (Hilty 2018). Morphometric characters, wing chord 52.167 mm, tail length 28 mm and bill depth 4.117 mm.

##### Geographical distribution.

Through Gulf slope of Mexico from Nuevo Leon, S Tamaulipas and E San Luis Potosí to N Chiapas, Yucatan Peninsula, E of Guatemala, Belize to N Honduras; in the Pacific slope from W Oaxaca, Mexico to NW Costa Rica (Hilty 2018).

#### Euphonia godmani

Taxon classificationAnimaliaPasseriformesFringillidae

Brewster 1889 stat. nov.

574F8B5D-3A64-5496-B4C2-24678FB5532D

Euphonia godmani Brewster, 1889, *Auk* 6: 90.
Tanagra affinis godmani ; Miller et al. 1957, *Cooper Ornithol. Soc. Pac. Coast Avifauna* 33: 298.
Euphonia affinis godmani ; Dickerman, 1981, *Occ. Pap. Mus. Zool. Louisiana State. Univ*. 59: 1.

##### Morphological characterization.

Male. Very similar to *E. affinis* with white undertail coverts feathers (Hilty 2018). Morphometric characters, wing chord 54.613 mm, tail length 27.333 mm and bill depth 4.683 mm. Female. Paler respect to *E. affinis* and with white belly and with undertail coverts feathers (Hilty 2018). Morphometric characters, wing chord 53.833 mm, tail length 27.667 mm and bill depth 4.327 mm.

Geographical distribution: Along to Pacific slope of Mexico from SE Sonora S to C Guerrero (Hilty 2018).

## Discussion

We provide molecular, morphological, behavioral, and environmental niche evidence supporting the existence of two evolutionary lineages within the *Euphonia affinis* complex (*E. godmani* and *E. affinis*). De Queiroz (2007) proposed that different types of data (morphological, ethological, ecological, molecular, etc.) can support species delimitation, if the lineages under study are evolving separately and can then be considered different species. Therefore, in our study we show these lineages have disjunct distributions, they have different plumages (white undertail coverts in *E. godmani*, and yellow undertail coverts in *E. affinis*, Hilty 2018), and we found significant differences in morphometric measurements. We did not find evidence of a third lineage corresponding to the subspecies *E. affinis olmecorum* proposed by Dickerman (1981); rather, it represents intraspecific variation within *E. affinis*.

### Phylogenetics and genetic variation

We found three lines of evidence in the molecular data to support the taxonomic split of the *E. affinis* complex at species level. The first is the reciprocal monophyly between *E. affinis* and *E. godmani* found in the multilocus analysis using mitochondrial and nuclear genes, where the samples of *E. affinis olmecorum* are included into *E. affinis*. These results agree with the proposals by Ridgway and Friedman (1901) and the taxonomic proposal of Navarro-Sigüenza and Peterson (2004). The second line of evidence is the genetic distance between the western and eastern groups, which is similar to other *Euphonia* species close to the *E. affinis* complex (Table 2). These genetic distance values are also similar to distances found in other bird complexes distributed in Mexico and Central America that have been recognized as distinct species (Puebla-Olivares et al. 2008, Arbeláez-Cortés and Navarro-Sigüenza 2013, Zamudio-Beltrán and Hernández-Baños 2015). The third line of evidence is the high index of genetic fixation FST (Table 2) and the haplogroups in the haplotype networks (Fig. 1). It is important to mention that the haplotype networks showed geographical correspondence, with *E. godmani* in western Mexico, and *E. affinis* in eastern Mexico and Central America. These results are consistent with studies of other bird species distributed in Mesoamerica (Smith et al. 2011, Ramírez-Barrera et al. 2018).

### Morphometrics

Our analysis revealed significant differences between *E. a. godmani* and *E. a. affinis* in six characters among lineages. Bill Depth and Wing Chord are bigger for *E. a. godmani*, while *E. a. affinis* has bigger dimensions on Tail Length. Even though the rest of characters have significant differences, in the PCA plots the Tail Length, Bill Length and Bill Width characters do not show dispersion between both lineages, so we can assign Wing Chord, Tail Lenth and Bill Depth as diagnostic characters for males, and Wing Chord and Bill Depth as diagnostic characters for females. These results are similar to *Phaethornis mexicanus* morphometric patterns, a species also distributed along the Atlantic and Pacific Slope, where the Pacific lineage also shows bigger dimensions vs. the Atlantic lineage (Arbeláez-Cortés and Navarro-Sigüenza, 2013).

### Vocalization

Our results show that there are significant differences in the temporal characteristics of calls between *E. godmani* and *E. affinis* while we found that there is little divergence in spectral structure or frequency measurements. *E. godmani* emits call notes at a significantly faster rate than *E. affinis*. Many bird species are highly sensitive to temporal cues in recognizing conspecific vocalizations (Dooling and Prior, 2016), which suggests that while call structure and frequency in this complex has been conserved, variation in tempo could be an important cue in conspecific recognition.

### Ecological niche similarity

*Euphonia affinis* and *E. godmani* represent two different lineages with no significant conservatism in their ecological niches (west vs. east). The env-PCA, also, showed a larger ENM for *E affinis*, respect to *E. godmani*, also the western lineage has a limited ability to predict its ENM in the geographic area of *E. affinis*. The western coast of Mexico is characterized by a highly contrasting dry season vs. a wet season over the year, this characteristic is unique with respect to the eastern tropical area, so *E. godmani* has become restricted to these conditions. These results are similar to other taxa with sister lineages distributed along the Pacific and Atlantic slopes in Mesoamerican (Hernández-Canchola and León-Paniagua, 2017). It is interesting that *E. godmani* shows a reduction in ecological niche, while *E. affinis* presents a broader ecological niche. That may suggest a scenario where *E. godmani* was able to invade the western area of Mexico, and, in the absence of ecological competition from other Euphonias, it adapted and specialized to the floristic resources, as well as to the temperature and precipitation conditions of the area. While *E. affinis* conserved a broader ecological niche, as reflected in its geographical distribution, allowed it to explore more regions and resources, even in the presence of different species of Euphoniinae.

### Biogeographical history

Lineage divergence between *E. godmani* (western Mexico) and *E. affinis* (eastern Mexico and Central America) occurred ~ 2.6 Mya (1.5–4.0 Mya HPD 95%), a range between the Pliocene and Pleistocene epochs. During the Pliocene, the Sierra Madre Occidental and the Transmexican Volcanic Belt finished emerging, which made the Pacific Slope drier than the Atlantic slope, due to the hillside effect (Graham and Dilcher, 1995). Additionally, the drier conditions were favored by meteorological phenomena that made the Pacific coast warmer than the Atlantic coast in the northern hemisphere (Molnar and Cane 2007). These events were decisive for the conformation of the tropical deciduous forest that extended throughout the Pacific from western Mexico to western Panama. According to studies of paleontological and molecular evolution, botanical elements present in the dry forests today were already present in said area since the Miocene (Graham and Dilcher 1995, [Bibr B9][Bibr B20]) however, from the Middle Pliocene to the late Pliocene these elements were unified as a plant community, promoting the diversification of some botanical groups (Becerra et al. 2005, de-Nova et al. 2012). Also, significant isolated periods of dry forest have been attributed to diversification in the Pacific Slope area (Becerra 2005, de-Nova et al. 2012, Willis et al. 2015). As a consequence, this province is characterized by a pattern of a high number of endemic lineages and species (Zaldívar et al. 2004, García-Deras et al. 2007, Zarza et al. 2008, Ramírez-Barrera et al. 2018). We found two threads of evidence that support the relationship between divergence of lineages for *E. affinis* and the origin of dry forests. The first evidence is the age of 2.6 Mya when the West and East lineages diverged during the late Pliocene, which coincides with the establishment of dry forests in Western Mexico. The other evidence is the adaptation and restriction of the environmental niche of *E. a. godmani* to the environmental conditions of Western Mexico. Other biogeographic events of Mesoamerica that shaped the biota were the closure of the Isthmus of Panama during the late Pliocene and the orographic changes in the Atlantic slope by the last raise of Transmexican Volcanic Belt and the Sierra Madre Oriental. However, the Atlantic Slope shows a wide mosaic of environments and ecosystems (Graham and Dilcher 1995), in contrast to the dry forest-dominated West slope, which could explain the more extensive environmental niche of *E. a. affinis*.

In addition to the consequences of the orographic changes of the Pliocene, during the Late Pliocene, global and continuous cooling periods were frequent, and during the Pleistocene the climatic oscillations were defined by glacial and interglacial periods (Zachos et al. 2001). During the glaciations, the species inhabiting temperate zones expanded their distribution to lower altitudes (Moreno-Letelier et al. 2014), while the geographic distribution of tropical vegetation was reduced. Tropical forests were affected by periods of low humidity which favored the reduction of the distributional range of several species, thus probably promoting speciation in plant species (Gentry 1982) as well as in the fauna of these forests, including birds (Smith and Klicka 2010). The divergence between Mesoamerican lowlands species has been attributed to these climatic changes, for example, amphibians (Greenbaum et al. 2011), reptiles (Ruane et al. 2014), mammals (Castañeda-Rico et al. 2014), and birds (Arbeláez-Cortés and Navarro-Sigüenza 2013). This work shows that orographic and environmental changes promoted the divergence of two lineages within *E. affinis*, probably due to isolation events and environmental adaptations, which in turn could accentuate the present differences in morphological, genetic, behavioral, and ecological characteristics previously described.

## Conclusions

We incorporated different kinds of information to help us identify lineages within the *Euphonia affinis* species complex and understand the speciation process (De Queiroz 2005, 2007, 2011, Padial et al. 2010). We have demonstrated a sharp genetic split between *E. a. affinis* and *E. a. godmani* and we found a similar pattern in morphometrics, vocalizations, as well as in ecological niche data. So, we can conclude that our data support the consideration of *E. affinis* and *E. godmani* as two species.

## Supplementary Material

XML Treatment for Euphonia affinis

XML Treatment for Euphonia godmani

## Supplementary materials

Supplementary material 1 Tables S1, S2, S3. Sampling, genbank sequences and sequences of primers Data type species data https://binary.pensoft.net/file/435160This dataset is made available under the Open Database License (http://opendatacommons.org/licenses/odbl/1.0/). The Open Database License (ODbL) is a license agreement intended to allow users to freely share, modify, and use this Dataset while maintaining this same freedom for others, provided that the original source and author(s) are credited.Melisa Vázquez-López, Blanca E. Hernández-Baños

Supplementary material 2 Raw morphometric data and collection information Data type morphological data https://binary.pensoft.net/file/435161This dataset is made available under the Open Database License (http://opendatacommons.org/licenses/odbl/1.0/). The Open Database License (ODbL) is a license agreement intended to allow users to freely share, modify, and use this Dataset while maintaining this same freedom for others, provided that the original source and author(s) are credited.Melisa Vázquez-López and Blanca E. Hernández-Baños
