# Supplementary material for: Multilocus, phenotypic, behavioral, and ecological niche analyses provide evidence for two species within Euphonia affinis (Aves, Fringillidae)
Source: Zookeys. 2020 Jul 23;952:129–57. doi: 10.3897/zookeys.952.51785 (PMC7394775; doi:10.3897/zookeys.952.51785)
Supplement: Supplementary material 1 — Tables S1, S2, S3. Sampling, genbank sequences and sequences of primers [file zookeys-952-129-s001.docx]

| **Table S1. Tissues samples of *E. affinis* and outgroup.** | | | | | | | | | | | |
| --- | --- | --- | --- | --- | --- | --- | --- | --- | --- | --- | --- |
| Species | Catalogue # | Locality | | Latitude | | Longitude | | | Institution | |  |
| *1. E. a. godmani* | VGR 1073 | Bajosori 10 km SO of Choix, Ejido 1. Municipio Choix, Sinaloa, Mexico. | | 26.65 N | | -108.9 W | | | MZFC | |  |
| *2. E. a. godmani* | VGR 1189 | Bajosori 10 km SO of Choix, Ejido 1. Municipio Choix, Sinaloa, Mexico. | | 26.65 N | | -108.9 W | | | MZFC | |  |
| *3. E. a. godmani* | ORT 003 | 3.5 km to Singayta. San Blas, Nayarit, Mexico. | | 21.6031 N | | -105.2377 W | | | MZFC | |  |
| *4. E. a. godmani* | CPM 003 | .Mpio. El Tuito, Rancho "Los Cuates".Jalisco, Mexico. | | 20.296257 N | | -105.393 W | | | MZFC | |  |
| *5. E. a. affinis* | CONACYT 1301 | Nizanda Road to Sábanas. Municipio Asunción Ixtaltepec, Oaxaca, Mexico. | | 16.677953 N | | -95.02317 W | | | MZFC | |  |
| *6. E. a. affinis* | GES 350 | El Arroyo, 6 Km to S of Silvituc. Campeche, Mexico. | | 18.5927 N | | -90.2561 W | | | MZFC | |  |
| *7. E. affinis* | MOL 15_58 | Km 191 road of Oaxaca – Puerto Angel, Santiago La Galera. Candelaria Loxicha, Oaxaca; Mexico. | | 15.971389 N | | -96.478056 W | | | MZFC | |  |
| *8. E. a. affinis* | NIZA 120 | Nizanda, Piedra Azul. Asuncion Ixtaltepec, Oaxaca, Mexico. | | 16.7452777 N | | -95.115 W | | | MZFC | |  |
| *9. E. a. affinis* | Y 408 192 | Rancho San Salvador, Anexa Chunkilin. Rio Lagartos, Yucatan, Mexico. | | 21.5095 N | | -88.0298 W | | | MZFC | |  |
| *10. E. a. affinis* | YUC 13-60 | Reserva de la Biosfera Rio Lagartos. Tizimin, Yucatan, Mexico. | | 21.56292 N | | -88.07627 W | | | MZFC | |  |
| *11. E. a. affinis* | MAGH 10_25 | Reserva de la Sierra Gorda, El Pantanal, Orilla del Rio Sta. Maria m 676. | | 21.45516 N | | -99.84321 W | | | CNAV/IB | |  |
| *12. E. a. affinis* | MAGH 10_36 | Reserva de la Sierra Gorda, El Pantanal, Orilla del Rio Sta. Maria m 676. | | 21.45516 N | | -99.84321 W | | | CNAV/IB | |  |
| *13. E. a. affinis* | MAGH 08_201 | Reserva de la Sierra Gorda, El Pantanal, Orilla del Rio Sta. Maria m 676. | | 21.45516 N | | -99.84321 W | | | CNAV/IB | |  |
| *14. E. a. affinis* | PEP 2770 | Mountain San Martín Pajapan 2 Km to S La Valentina. Veracruz, Mexico. | | 18.288 N | | -94.717 W | | | CNAV/IB | |  |
| *15. E. a. affinis* | PEP 2769 | Mountain San Martín Pajapan 2 Km to S La Valentina. Veracruz, Mexico. | | 18.288 N | | -94.717 W | | | CNAV/IB | |  |
| *16. E. a. affinis* | PEP 2768 | Mountain San Martín Pajapan 2 Km to S La Valentina. Veracruz, Mexico. | | 18.288 N | | -94.717 W | | | CNAV/IB | |  |
| *17. E. a. affinis* | PEP 2602 | Sierra de Santa Martha 5km to NE of Bastonal. Mexico. | | 18.354 N | | -94.927 W | | | CNAV/IB | |  |
| *18. E. a. affinis* | DAB 1922 | Las Plazulas, Laguna Blanca. Departament of Granada, Nicaragua. | | 11.766 N | | -85.995 W | | | UWBM | |  |
| *19. E. a. affinis* | GAV 2440 | San Felipe Retalhuleu 5 Km to S, Finca el Niño. Departament of Retalhuleu, Guatemala. | | 14.6 N | | 91.611 W | | | UWBM | |  |
| *20. E. a. affinis* | 2099 | 24 km to S of Silvituc. Campeche, Mexico. | | 18.2333 N | | -90.2 W | | | KU | |  |
| *21. E. a. affinis* | 1983 | 24 km to S of Silvituc. Campeche, Mexico. | | 18.2333 N | | -90.2 W | | | KU | |  |
| *22. E. a. affinis* | 9347 | Animas. Zacatecoluca. La Paz, El Salvador. | | 13.33 N | | -88.85 W | | | KU | |  |
| *23. E. a. affinis* | 9346 | Animas. Zacatecoluca. La Paz, El Salvador. | | 13.33 N | | -88.85 W | | | KU | |  |
| **Outgroup** | | | | | | | | | | | |
| *24. Chlorophonia occipitalis* | | | DAB 1391 | | 10 km N of Matagalpa, Nicaragua. | | 13.015 N | -85.9233 W | | UWBM | |
| *25. E. chlorotica* | | | 3226 | | 14 km W of Bahia Negra, Estancia Triunfo; Alto Paraguay, Paraguay. | | -20.26 N | -58.2666667 W | | KU | |
| *26. E. chlorotica* | | | 3199 | | 14 km W of Bahia Negra, Estancia Triunfo; Alto Paraguay, Paraguay. | | -20.26 N | 58.2666667 W | | KU | |
| *27. E. luteicapilla* | | | B 28445 | | 2 km SO of Río Farfan; Panama, Panama | | 999 | 999 | | LUSMZ | |
| *28. Haemorhous mexicanus* | | | QRO. 251 | | La Florida, Careyta de Montes, Queretaro, Mexico. | | 20.856667 N | -99.792778 W | | MZFC | |
| MZFC: Museo de Zoología Alfonso L. Herrera, Facultad de Ciencias, UNAM. CNAV/IB: Colección Nacional de Aves/Instituto de Biología, UNAM. UWBM: University of Washington, Burke Museum, USA. KU: University of Kansas, USA. LUSMZ: Museum of Natural History of Louisiana, USA. | | | | | | | | | | | |

| Table S2.  **GenBank sequences used for the outgroup.** | | |
| --- | --- | --- |
| Species | Accession number | Author |
| *Fringilla montifringilla*  *Fringilla teydea*  *Haemorhous mexicanus* | GU816920 (ODC)  GU816851 (ND2)  JN715184 (GAPDH)  EU739811 (MUSK  KC767747 (BRM)  KC292816 (ND2)  KC292792 (MUSK)  KM112839 (ODC)  JN715155 (GAPDH) | Fjeldsa *et al.*(2010)  Fjeldsa *et al.*(2010)  Zuccon *et al.*(2011)  Hackett (2008)  Lifjeld *et al.*(2013)  Smith *et al.*(2013)  Smith *et al.*(2013)  Smith *et al.*(2013)  Smith *et al.*(2013) |
| *Carpodacus vinaceus* | HQ284789 (GAPDH)  KJ455737 (ODC)  JN715444 (ND2) | Topfer *et al.*(2011)  Priece *et al.*(2014)  Priece *et al.*(2014) |
| *Euphonia finschi*  *Euphonia chlorotica*  *Euphonia musica*  *Euphonia violacea*  *Euphonia lanirostris*  *Euphonia xanthogaster*  *Euphonia minuta* | AF290106 (ND2)  JN15362 (ODC)  JN715180 (GAPDH)  JN7151175 (GAPDH)  JN715451 (ND2)  JN15360 (ODC)  JN715453 (ND2)  JN715363 (ODC)  JN71581 (GAPDH)  JN715449 (ND2)  JN715358 (ODC)  JN715176 (GAPDH)  JN715454 (ND2)  JN715364 (ODC)  JN715450 (ND2)  JN715359 (ODC)  JN715177 (GAPDH) | Zuccon *et al.*(2011)  Zuccon *et al.*(2011)  Zuccon *et al.*(2011)  Zuccon *et al.*(2011)  Zuccon *et al.*(2011)  Zuccon *et al.*(2011)  Zuccon *et al.*(2011)  Zuccon *et al.*(2011)  Zuccon *et al.*(2011)  Zuccon *et al.*(2011)  Zuccon *et al.*(2011)  Zuccon *et al.*(2011)  Zuccon *et al.*(2011)  Zuccon *et al.*(2011)  Zuccon *et al.*(2011)  Zuccon *et al.*(2011)  Zuccon *et al.*(2011) |

| **Table S3. PCR, sequencing primers, sequence lengths, and evolution fit models.** | | | | |
| --- | --- | --- | --- | --- |
| **GENE** | PRIMER | Annealing temperature | Sequence Length | Fit Model |
| **ND2^1^** | ^1^L5219 CCC ATA CCC CGA AAA TGA TGC CCA TAC CCC GAA AAT GAT G  H6313 CTC TTA TTT AAG GCT TTG AAG GC | 54°C | 1044 | TVM+G |
| **ODC^2^** | ^2^ODC F GTT CTA CTT TCT CTG GAA CTA CTG CTC  ODC R CAT CAA GTC AGT GAC TTA GCT AGC | 65°C | 557 | HKY |
| **GAPDH^3^** | ^3^GAPDH 890 intron 11 ACC TTT AAT GCG GGT GCT GGC ATT GC  GAPDH 950 intron 12 CAT CAA GTC CAC AAC ACG GTT GCT GTA | 55°C | 283 | HKY |
| **BRM^4^** | ^4^BRM_intron 11 Mex15 AGC ACC TTT GAA CAG TGG TT  BRM_intron 11 Mex16 TAC TTT ATG GAG ACG ACG GA | 55°C | 314 | HKY |
| **MUSK^5^** | ^5^MUSK F CTT CCA TGC ACT ACA ATG GGA AA  MUSK R CTC TGA ACA TTG TGG ATC CTC AA | 58°C | 500 | TPM1uf |
| ^1^NADH Dehydrogenase Subunit 2, Sorenson et al. 1999. ^2^Ornithine Decarboxylase, Allen and Omland (2003).^3^Glyceraldehyde-3-phosphate dehydrogenase intron 11, Friesen et al. 1997. ^4^BRM transcription regulatory protein intron 15, Marthinsen et al. 2009. ^5^Muscle, skeletal receptor tyrosine-protein kinase, Kimball et al. 2009. | | | | |
